# Severe Osteomyelitis and Septic Arthritis due to *Serratia marcescens* in an Immunocompetent Patient

**DOI:** 10.1155/2015/347652

**Published:** 2015-06-15

**Authors:** Hiba Hadid, Muhammad Usman, Sudeep Thapa

**Affiliations:** Department of Internal Medicine, Henry Ford Hospital, 2799 W. Grand Boulevard, Detroit, MI 48202, USA

## Abstract

Septic arthritis and osteomyelitis due to *Serratia marcescens* in immunocompetent patients without risk factors are extremely rare. Here, we report a case of septic arthritis and severe adjacent osteomyelitis of the tibia due to *Serratia marcescens* in a diabetic community-dweller patient. The patient had no contact with healthcare workers or facilities and had no chronic disease except for poorly controlled diabetes. Without predisposing risk factors, this type of infection is extremely rare, even in diabetics.

## 1. Introduction

Osteomyelitis is an infection localized to bone. Septic arthritis is an infection localized to the jont. Both of these infections can occur as a result of contiguous spread of infection to/from adjacent tissue, as a result of direct inoculation through trauma or surgery or as a result of hematogenous spread from other distant infected sites. Most common pathogens causing these infections are* Staphylococcus aureus*, coagulase-negative staphylococci, and aerobic Gram-negative bacilli [1]. Other less common organisms are enterococci, streptococci, anaerobes, and fungi.* Serratia* species are Gram-negative facultative anaerobic bacilli of the Enterobacteriaceae group that are known to cause a spectrum of clinical diseases in humans including osteomyelitis and septic arthritis [2, 3]. Of this bacterial group,* Serratia marcescens* is the main human pathogen. Over the last few decades, it has been increasingly reported as a cause of opportunistic nosocomial infections. The most common infections due to this organism are respiratory and urinary tract infections. Other less common infections are endocarditis, bacteremia, peritonitis, and cellulitis [4, 5]. On the other hand, septic arthritis and osteomyelitis due to* Serratia marcescens* are very rare, especially in immunocompetent individuals.

## 2. Case Presentation

A 72-year-old male presented to our emergency department with distal right leg pain. He has a past medical history of uncontrolled type 2 diabetes, medication noncompliance, and coronary artery disease. His surgical history was also significant for a right distal fibular open fracture nine years ago requiring a combination of washout, open reduction with internal fixation. His postoperative course was uncomplicated and he had since returned to full and normal function. He had no history of infection or problems with his leg in the years thereafter. The patient presented to us with a seven-day history of worsening right ankle pain. He works barefoot for a significant amount of time in a golf course but did not recall any fall, trauma, animal or bug bites, skin laceration, or accidents. His vital signs were normal and his examination was remarkable only for tenderness in his right ankle. His ankle X-ray showed the hardware in proper position with only minor degenerative changes. The patient was discharged home without a definitive diagnosis and provided with pain medications. The patient presented to his primary care doctor for follow-up three days later with increased ankle pain, swelling, inability to bear weight, fevers, chills, and lightheadedness. He was found to be hypotensive with exam findings suspicious for septic arthritis. He was sent immediately to the hospital, where he was found to be in severe sepsis. He was hypotensive with blood pressure of 70/40, tachycardic at 112, and febrile at 39.0 degree Celsius. On labs, he had elevated creatinine from baseline, blood glucose of 400 mg/dL, and lactate of 4.2 mmol/L. He had no leukocytosis. His hemoglobin A1c was 12%. Blood cultures and urinalysis were obtained and the patient was started on early goal directed therapy with quick stabilization of his vital signs. Joint aspiration was performed revealing grossly blood-tinged thick fluid with 8600 WBC, with 97% neutrophils. His aspirate and blood cultures returned positive for* Serratia marcescens*. Broad-spectrum antibiotics were deescalated to Ertapenem. On hospital day 2, the patient was taken to the operating room for incision and washout. He was found to have significant purulent fluid in the tibiotalar joint. A drain was placed to aid in drainage. MRI was obtained at this time and showed findings consistent with osteomyelitis involving the distal tibia. There was also suspected sinus tract at the medial cortex just above the medial malleolus. On day 4, the patient underwent hardware removal with further washout performed. The implanted hardware in the distal fibula, although appeared uninfected, was removed. His tibia was incised with significant noted purulence and liquefaction of the bone extending proximally, requiring extensive washout and debridement. Despite the antibiotics and drainage, his joint remained purulent and appeared to have significant residual infection and inflammation requiring successive washouts on hospital days 6, 11, and 13. On hospital day 13, he also had antibiotic-impregnated beads placed. All of the cultures obtained from the joint remained positive. All repeat blood cultures remained negative. Since the most common source of* Serratia* is the urine and to rule out secondary sources leading to hematogenous spread, kidney and prostate ultrasounds were performed and were negative. Urinalysis was also noted to be negative on admission. The source of infection was believed to be inoculation through the skin from the golf-course soil. The patient's creatinine and vital signs recovered and the patient was discharged to subacute rehabilitation on day 14 on a six-week course of Ertapenem. On his 5-week follow-up, he was doing well on his antibiotics therapy with no systemic or localized signs of ongoing infection.

## 3. Discussion

*Serratia marcescens* is an opportunistic pathogenic Gram-negative bacillus that has emerged as a serious cause of nosocomial infections [1].* Serratia marcescens* species are capable of thriving on diverse environments including water and soil, but most commonly in healthcare settings. In the last few decades, it has become more common in intensive care units as a cause of bacteremia, pneumonia, and urinary tract infections [6–8]. Primary joint infections, however, secondary to* Serratia marcescens* are extremely rare. Almost all of the cases have occurred in either trauma patients, drug addicts, immunosuppressed patients, critically ill patients, those receiving joint injections, or perioperative patients [7–11]. Our patient did not have any of these risk factors neither did he have any contact with any healthcare facility for the year prior to the onset of his symptoms. The occurrence of this infection without any of the previously reported risk factors is extremely rare. It is important to note, however, that our patient had uncontrolled diabetes and having a hardware in his right ankle made it more vulnerable. Inoculation of the bacteria from the soil on the golf course into his skin and joint is the most likely cause.

There are some case reports of critically ill patients with septic joints secondary to hematogenous spread. These patients had either central catheters or a urinary tract infection with cultures positive for* Serratia* [12]. Septic joints in these cases were a secondary event. In our patient, although he was bacteremic on admission, he had no urinary tract infection, no open wounds, and no central catheters. It is believed that the septic joint was the primary event. That is supported by the extent of bone involvement and destruction noted on examination in the operating room. Since the patient was evaluated in the emergency department and then discharged home only to come several days later with similar symptoms, it is very unlikely that he was bacteremic all along without significant hemodynamic instability.* Serratia* septicemia is rapidly fatal if untreated. Treatment of septic arthritis and osteomyelitis includes a prolonged course of antibiotic therapy in addition to successive joint washouts [13–15].* Serratia marcescens* is very difficult to eradicate especially if there is significant bone destruction such as in our patient. Although our patient had several washouts and was on intravenous antibiotics, all of his joint cultures remained positive. The decision was to use antibiotic-impregnated beads which would ensure better delivery of the medication to the tissue involved than systemic therapy alone. Antibiotic beads combined with a prolonged course of intravenous antibiotics and very close follow-up would hopefully help eradicate this aggressive infection.
